# Periodontal Disease and Metabolic Syndrome in Women at Prior High Risk for Gestational Diabetes

**DOI:** 10.1002/cre2.70053

**Published:** 2024-12-12

**Authors:** Hanna Poulsen, Jukka H. Meurman, Hannu Kautiainen, Emilia Huvinen, Saila Koivusalo, Johan G. Eriksson

**Affiliations:** ^1^ Department of Oral and Maxillofacial Diseases University of Helsinki Helsinki Finland; ^2^ Department of General Practice and Primary Health Care University of Helsinki and Helsinki University Hospital Helsinki Finland; ^3^ Department of General Practice and Primary Health Care University of Helsinki Helsinki Finland; ^4^ Department of Obstetrics and Gynaecology University of Helsinki Helsinki Finland

**Keywords:** gestational diabetes, metabolic syndrome, periodontal disease

## Abstract

**Objectives:**

This study aimed to assess the association between periodontal disease and metabolic syndrome (MetS) among women at prior high risk for gestational diabetes with the hypothesis that women with MetS show more signs of periodontal disease than women without MetS.

**Material and Methods:**

A total of 112 women from an original study cohort of 348 women at high risk of gestational diabetes were examined 4–6 years postpartum. Diagnosis of MetS was based on the National Cholesterol Education Program Adult Treatment Panel III diagnostic criteria. Insulin resistance was approximated by the homeostatic model assessment for insulin resistance. Full‐mouth examinations and panoramic radiographs provided the total dental index, number of teeth, and decayed, missing, and filled teeth index. Clinical examination assessed bleeding on probing, probing depth, visible plaque index, signs of infection, and clinical attachment levels. The periodontal inflammatory burden index (PIBI) was also calculated. Information on oral health habits, symptoms, and individual opinions on oral health was collected through questionnaires.

**Results:**

Five years after delivery, 21% of the women had MetS, and they had more gingivitis compared to those without MetS (bleeding on probing: 52% and 44%, *p* = 0.011). Women with MetS tended to have more periodontitis than those without (39% and 25%, *p* = 0.13). A high PIBI correlated with insulin resistance (partial correlation of PIBI and homeostatic model assessment for insulin resistance: 0.25 *p* < 0.05).

**Conclusions:**

Periodontal disease was associated with insulin resistance and MetS in women at prior high risk of developing gestational diabetes.

## 1 Introduction

Oral diseases, in particular periodontal disease, cause low‐grade systemic inflammation, which is connected to numerous non‐communicable diseases such as type 2 diabetes (T2D), obesity, and metabolic syndrome (MetS) (Esser et al. 2014; Lamster and Pagan 2017; Saltiel and Olefsky 2017). A strong association between periodontal disease and diabetes, as well as milder degrees of hyperglycemia, has been reported, and a two‐way relationship exists (Bascones‐Martinez et al. 2011; Borgnakke et al. 2018; Janket et al. 2003; Kocher et al. 2018; Mattila et al. 1989; Sanz et al. 2018). Taking into account the close relationship between T2D and cardiovascular disease (CVD), it is not surprising that periodontal disease also increases the risk for CVD (Kebschull, Demmer, and Papapanou 2010; Sanz et al. 2020; Sen et al. 2024). The common underlying mediator is most probably chronic low‐grade inflammation (Sheiham and Watt 2000).

Due to the current epidemic of obesity and a sedentary lifestyle, MetS is a common and increasing global health concern (Alberti et al. 2009; Alberti, Zimmet, and Shaw 2006). It is characterized by hyperglycemia, abnormal lipid levels, hypertension, and central obesity (Alberti et al. 2009). These conditions are also interrelated risk factors for T2D and CVD (Eckel, Grundy, and Zimmet 2005; Alberti et al. 2009). A pre‐stage condition occurs before full‐blown MetS when it is still possible to influence the subject's metabolism and improve prognosis.

Gestational diabetes mellitus (GDM; i.e., glucose intolerance first diagnosed during pregnancy) can be considered an early marker of an increased risk for MetS (ElSayed et al. 2023). The dysglycemia associated with GDM also adversely affects periodontal tissues (Demmer et al. 2012). Periodontitis is linked with the development of GDM, and poor periodontal health relates to a greater risk of developing T2D (Abariga and Whitcomb 2016; Borgnakke et al. 2018; Sanz et al. 2018). Also, GDM elevates the risk for later development of T2D and CVD, independent of obesity status (Huvinen et al. 2018). Because all these conditions, including periodontal disease, associate with low‐grade inflammation, poor oral health has indeed been suggested to be a risk factor for MetS among women with prior GDM (Bullon et al. 2014). It is still unclear, however, why some women at high risk for GDM develop MetS and others do not. Some risk factors of MetS have been recognized, but the pathogenesis still remains unclear.

In the present study, we studied the potential association between periodontal disease and MetS in the early years after pregnancy among Finnish women with high GDM risk. We anticipated that poor oral health might also be associated with insulin resistance. The study hypothesis was that women with MetS show more signs of periodontal disease than women without MetS.

## 2 Materials and Methods

**Figure 1:**
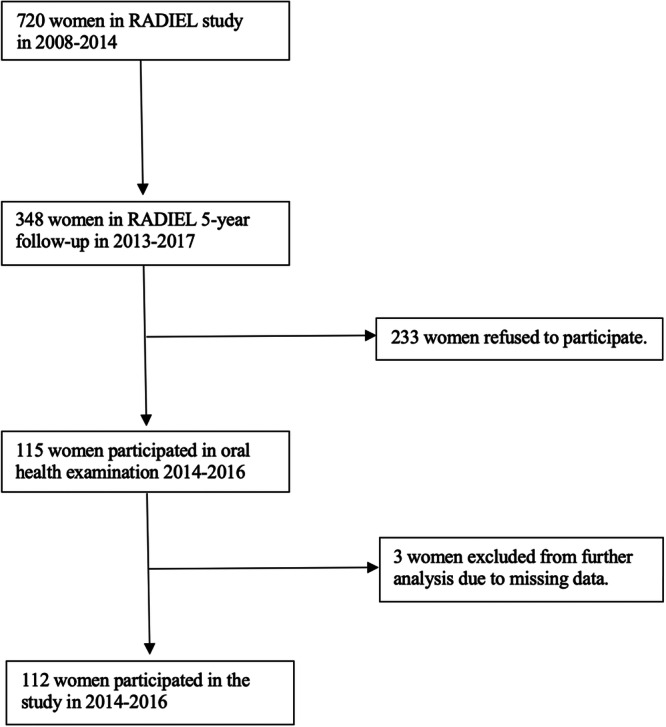
Study population.

This is a sub‐study of the Finnish Gestational Diabetes Prevention Study (RADIEL), and the publication by Rönö et al. (2014) provides details of the study design. The RADIEL study was a randomized controlled multi‐center intervention trial on 720 women recruited between the years 2008 and 2014. This study aimed to prevent GDM through lifestyle modifications (physical activity and diet). The women were recruited before their 20th gestational week. All women were at high GDM risk because of obesity (BMI ≥ 30 kg/m^2^), or because they had a history of GDM. The follow‐up study invited participants 4–6 years after delivery, and 348 of them responded. The present oral health investigation is a cross‐sectional study that includes 112 of these non‐pregnant women, aged 27–49 years, as shown in Figure 1.

An electronic stadiometer and an electronic scale assessed the participants’ weight and height, with accuracy to the nearest 0.1 kg and 0.1 cm, respectively. Waist circumference was measured to the nearest 0.5 cm. Questionnaire data were collected on education, smoking habits, and physical activity.

An overnight fast preceded venous blood sampling. Enzymatic hexokinase assay (Roche Diagnostics, Basel, Switzerland) was used to assess plasma glucose concentrations and low‐density lipoprotein (LDL), high‐density lipoprotein (HDL), triglycerides (TG), and total cholesterol were analyzed using routine methods in the hospital laboratory. Glycated hemoglobin (HbA_1c_) was assessed with an immunoturbidimetric analyzer. A 2 h, 75 g oral glucose tolerance test provided data on glucose tolerance. Insulin resistance and β‐cell function were estimated using HOMA‐IR and HOMA‐β indices from fasting insulin and fasting glucose concentrations, according to Matthews et al. (1985).

MetS was defined according to the National Cholesterol Education Program Adult Treatment Panel III (NCEP/ATP III) criteria (Alberti et al. 2009). The diagnosis was set when three of the following five criteria were fulfilled: waist circumference > 88 cm, systolic blood pressure > 130 mmHg or diastolic blood pressure > 85 mmHg, fasting plasma glucose ≥ 5.6 mmol/L, triglycerides ≥ 1.7 mmol/L, and HDL < 1.29 mmol/L.

The oral health sub‐study included a complete oral health examination and panoramic x‐rays of the jaw. A single examiner (H.P.) performed the examinations, uninformed of the participants' metabolic parameters. Data on the number of teeth, dental, periodontal, and mucosal health were registered. Bleeding on periodontal probing index (BOP) and visible plaque index (VPI) were examined from six surfaces of each tooth and calculated according to Ainamo and Bay (1975). Periodontal pocket depths were measured, and the presence of calculus was recorded from six surfaces of each tooth with a WHO probe. The enamel–dentin junction to the gingival margin defined gingival recession and adding pocket depth to that gave the clinical attachment loss (CAL). Periodontal disease was classified according to Eke et al. (2018) as mild, moderate, or severe (Eke et al. 2012). Total dental index (TDI), decayed, missing, and filled teeth (DMFT) index, and periodontal inflammatory burden index (PIBI), respectively, were determined (Mattila et al. 1989, Lindy et al. 2008). The panoramic x‐rays were analyzed for signs of infections in the teeth. Marginal alveolar bone loss was also recorded. A structured questionnaire collected data on women's oral health habits, potential symptoms, and the perception of their oral health. After the examination, all subjects received information on their oral health and x‐ray status. Women in need of oral health care were accordingly informed to have a dental appointment.

The descriptive data are presented as means with standard deviation (SD), as frequencies with percentages, or as medians with interquartile range (IQR). For between‐group comparisons, we used the *t*‐test and chi‐square test. In cases of violation of assumptions (e.g., non‐normality) for continuous variables, a bootstrap‐type method and Monte Carlo *p*‐values (for small numbers of observations) for categorical variables were used. Adjusted correlation (partial) coefficients PIBI values and the women's status of glucose metabolism and obesity were calculated using the Pearson method, with Sidak‐adjusted (multiplicity) probabilities. To examine the normal distribution of the variables, we used graphical evaluation and the Shapiro–Wilk test. STATA software, version 17.0 (StataCorp LP, College Station, TX) was used for all analyses.

The original RADIEL study was approved by the Ethical Boards of Helsinki University Central Hospital (Dnro 300/E9/06; September 14, 2006) and South Karelia Central Hospital (Dnro M06/08; September 11, 2008).

The current follow‐up study was further approved by the Ethical Committee of Obstetrics of the Hospital District of Helsinki and Uusimaa (TYH2013402; January 2014, 9). The oral health study section was further approved by the Ethical Committee of Women, Children, and Psychiatrics of Helsinki University Hospital (136/13/03/03/2014; April 24, 2014). All participants provided written informed consent before participating in the study.

## 3 Results

**Figure 2:**
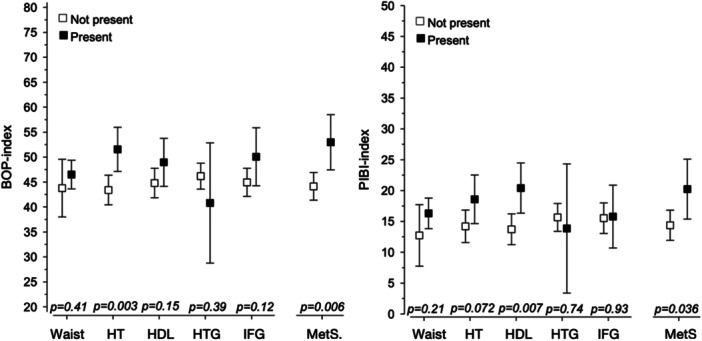
Associations between bleeding on probing (BOP) and periodontal inflammatory burden index (PIBI) with metabolic syndrome (MetS) and its individual components, respectively, adjusted for age, smoking status, and level of education (*N* = 112, *n* = 23 with MetS). Waist, waist circumference > 88 cm. HT, hypertension: systolic blood pressure > 130 mmHg or diastolic blood pressure > 85 mmHg. HDL, high‐density lipoprotein concentration (hyperlipidemia), below 1.29 mmol/L (50 mg/dL). HTG, hypertriglyceridemia, concentration of total triglycerides (hyperlipidemia) 1.7 mmol/L (150 mg/dL) or higher. IFG, impaired fasting glucose level from 5.6 mmol/L (100 mg/dL) to 6.9 mmol/L (125 mg/dL) (dysglycemia).

About one‐fifth of the women (23 of 112) with a prior high risk for GDM fulfilled the MetS criteria at the 5‐year follow‐up examination, as shown in Table 1. Of the oral health parameters, only gingivitis crude scores were significantly different between women with MetS compared to those without MetS, as shown in Table 2 (BOP: 52% versus 44%, *p* = 0.011). However, when adjusted for age, smoking status, and level of education, the women with MetS had significantly higher PIBI scores (*p* = 0.036) than the women without MetS. Significant differences were also detected in blood pressure with respect to BOP index scores, whereas PIBI scores, in turn, were associated significantly with blood pressure and HDL cholesterol (Figure 2).

**Table 1:** Basic characteristics of the subjects at 5‐year follow‐up.

Variable	ALL, *N* = 112	Non‐MetS, *n* = 89	MetS, *n* = 23
Age (years), mean (SD)	39.3 (5.1)	39.1 (5.1)	40.3 (5.2)
Weight (kg), mean (SD)	86.8 (19.9)	82.5 (18.6)	103.1 (16.3)
BMI (kg/m^2^), mean (SD)	31.4 (6.8)	30.0 (6.6)	36.7 (4.6)
Waist (cm), mean (SD)	104 (17)	100 (16)	117 (12)
Body fat (%), mean (SD)	38.1 (9.5)	36.4 (9.7)	44.6 (5.3)
Education years, mean (SD)	14.9 (1.9)	15.0 (1.8)	14.5 (2.0)
Smoking, *n* (%)	13 (12)	13 (15)	0 (0)
Physical activity (min/week), median (IQR)	60 (30−150)	90 (40−180)	60 (30−90)
HbA1c (%), mean (SD)	5.51 (0.30)	5.47 (0.30)	5.67 (0.27)
Fasting plasma glucose (mmol/L),^a^ mean (SD)	5.15 (0.55)	5.04 (0.50)	5.54 (0.57)
Insulin (mU/L)	10.22 (6.53)	8.92 (5.50)	15.23 (7.80)
HOMA‐IR	2.39 (1.65)	2.04 (1.38)	3.72 (1.95)
HOMA‐β	128 (76)	119 (68)	164 (94)
Total cholesterol (mmol/L),^b^ mean (SD)	4.59 (0.74)	4.63 (0.73)	4.44 (0.75)
HDL cholesterol (mmol/L),^b^ mean (SD)	1.54 (0.38)	1.63 (0.35)	1.21 (0.29)
LDL cholesterol (mmol/L),^b^ mean (SD)	2.91 (0.70)	2.90 (0.71)	2.97 (0.65)
Total triglycerides (mmol/L),^c^ mean (SD)	0.89 (0.38)	0.82 (0.33)	1.17 (0.44)
High‐sensitivity CRP (mmol/L), mean (SD)	2.54 (4.03)	1.96 (2.51)	4.79 (7.07)
Impaired glucose regulation, *n* (%)			
Impaired fasting glucose (IFG), *n* (%)	5 (4)	4 (4)	1(4)
Impaired glucose tolerance (IGT), *n* (%)	7 (6)	4 (4)	3 (13)
Diabetes mellitus (DM), *n* (%)	4 (4)	1 (1)	3 (13)
Blood pressure, (mmHg)			
Systolic	125 (13)	118 (12)	128 (13)
Diastolic	79 (9)	76 (8)	89 (7)

Abbreviations: MetS, metabolic syndome; BMI, body mass index, weight in kg/height in meters^2^; CRP, C‐reactive protein; HbA1c, glycated hemoglobin; HDL, high‐density lipoprotein; HOMA‐IR, homeostasis model assessment of insulin resistance; HOMA‐β, homeostasis model assessment of β‐cell function; IQR, interquartile range; LDL, low‐density lipoprotein; SD, standard deviation.

^a^ To get from mmol/L to mg/dL, multiply by 18.

^b^ To get from mmol/L to mg/dL, multiply by 38.67.

^c^ To get from mmol/L to mg/dL, multiply by 88.57.

**Table 2:** Oral health variables of the women (*N* = 112) without and with metabolic syndrome (MetS).

	Non‐MetS, *n* = 89	MetS, *n* = 23	*p‐*value
Total dental index (TDI), mean (SD)	1.65 (0.83)	1.96 (0.88)	0.16
Decayed, missing, and filled teeth (DMFT), mean (SD)	9.45 (5.20)	8.78 (5.86)	0.61
Periodontal inflammatory burden index (PIBI), mean (SD)	14.7 (11.8)	19.1 (13.5)	0.14
Bleeding on probing (BOP) %, mean (SD)	44.3 (13.4)	52.2 (12.2)	0.011
Visible plaque index (VPI) %, mean (SD)	0.14 (0.11)	0.19 (0.15)	0.14
Tooth brushing twice a day, *n* (%)	67 (75)	16 (70)	0.46
Interdental cleaning, *n* (%)	57 (64)	13 (57)	0.43
Periodontitis, *n* (%)			0.13
No periodontitis	15 (17)	2 (9)	
Mild periodontitis	52 (58)	12 (52)	
Moderate or severe periodontitis	22 (25)	9 (39)	

Abbreviation: SD, standard deviation.

Figure 2 also shows the periodontal parameters, BOP and PIBI scores, with respect to the health variables studied. Furthermore, there was a correlation between HOMA‐IR and the periodontal index scores, BOP and PIBI, respectively (Table 3).

**Table 3:** Results from partial correlation analyses of bleeding on probing and periodontal inflammatory burden index values with respect to the subjects’ status of glucose metabolism and obesity, adjusted with age, education, and smoking status.

	BOPr (95% CI)	PIBI
HOMA‐IR	−0.02 (−0.21 to 0.16)	0.25 (0.07 to 0.42)*
HOMA‐β	−0.05 (−0.23 to 0.14)	0.20 (0.02 to 0.38)
Insulin (mU/L)	−0.03 (−0.22 to 0.15)	0.26 (0.08 to 0.42)*
Body fat %,	0.09 (−0.10 to 0.27)	0.18 (−0.01 to 0.35)

Abbreviations: BOP, bleeding on probing; HOMA‐β, homeostasis model assessment of β‐cell function; HOMA‐IR, homeostasis model assessment of insulin resistance; PIBI, Periodontal Inflammatory Burden Index.

* *p* < 0.05; Sidak adjusted.

With regard to oral health habits, women with MetS reported brushing their teeth and cleaning their interdental spaces as frequently as those without MetS. Nevertheless, those with MetS had worse outcomes in all oral health variables, except in the DMFT index scores; however, the differences between the groups were not significant (Table 2).

## 4 Discussion

To the best of our knowledge, this is the first study focusing on oral health and MetS in the early years after pregnancy among women with high GDM risk. The study demonstrated that periodontal diseases were indeed more prevalent among high GDM risk women with MetS in the early years after pregnancy compared to those without MetS. Both the BOP and PIBI scores that were used as markers of periodontal disease correlated with MetS. We also found that periodontal inflammatory burden as measured with the PIBI scores correlated with insulin resistance. Thus, these results confirmed our study hypothesis. However, interestingly, oral health habits showed no difference compared to women with and without MetS. Nevertheless, our data indicate that periodontal diseases did associate with MetS regardless of oral health habits among these high‐risk women.

Insulin resistance and oral health have been studied previously. Andriankaja et al. (2018) observed in their longitudinal 3‐year study that insulin resistance approximated by the HOMA‐IR index was an independent predictor for periodontal disease. However, from a Hispanic population, their subjects were primarily male and at high risk for insulin resistance. Therefore, their results cannot be directly compared with ours. Demmer et al. (2012) studied non‐diabetic adults of both sexes and described an association between periodontal disease and insulin resistance. Similarly, our study showed that high PIBI scores are associated with HOMA‐IR index values. This supports the previous findings that there is a connection between periodontal disease and insulin resistance among obese women at high risk for MetS.

An association between periodontitis and MetS was also reported by Nibali et al. (2013) in their meta‐analysis and, similarly, Jepsen, Suvan, and Deschner (2020) concluded in their review article that there is an association between periodontitis, MetS, and obesity. Also, Nascimento et al. (2019) observed an association between MetS and periodontitis; however, in their study, it only applied to moderate to severe periodontitis. Our data support all these previous findings and show an association between periodontal disease and MetS among these high‐risk women. The connection between obesity and periodontal disease might indeed be bidirectional (Jepsen, Suvan, and Deschner 2020; Levine 2013).

The findings here discussed are of high clinical importance even though causality cannot be confirmed. Our study was the first of its kind by focusing on women with high risk or history of GDM. Hence, according to the present and previous data, we suggest that periodontal disease, regardless of its severity, could be a sign or a risk factor for MetS among obese and high‐risk GDM women. The present findings emphasize the importance of oral health care for these women.

The local chronic infection caused by periodontal disease increases the levels of cytokines and inflammatory mediators, leading to systemic subclinical, low‐grade inflammation. This, in turn, leads to the upregulation of many inflammatory mediators such as interleukin IL‐1b and IL‐6, tumor necrosis factor alpha (TNF‐a), and C‐reactive protein (CRP). All these factors may then affect detrimentally systemic conditions such as diabetes and MetS (Meurman and Bascones‐Martinez 2021). These and corresponding pathomechanisms may explain our results, emphasizing the need for careful monitoring of oral health in the early years after pregnancy, particularly among women who are at risk for GDM or MetS.

One of the strengths of our study was the thorough oral health examination, including full‐mouth panoramic x‐rays of the women. The fact that the examinations were performed by a single professional was a further strength as between‐examiners variability was thus avoided. Another strength is the thorough metabolic evaluation of our subjects. Furthermore, all women were from a homogenous Caucasian population in Finland. However, there are some limitations in our study. We did not have information about the subjects' oral health status at the time of the index pregnancy 5 years earlier. This is a weakness as we are not able to perform longitudinal analyses or confirm any causality. Neither do we have medical data annually recorded, so it is not clear when the subjects developed MetS. This is another limitation. Our recruitment criteria limit the generalizability of our results; however, it can also be viewed as a strength, as, to our understanding, we were the first to focus on this high‐risk group of women. Indeed, our findings give insight only into this group of women, and the results are not generalizable to other populations. Furthermore, the sample size was modest because many women refused to participate in the oral health examination.

Nevertheless, as emphasized before, the results confirmed our hypothesis by showing that women with MetS appeared more likely to also have more gingivitis than those who had not MetS. It is important to keep in mind that poor oral health seems to be linked to insulin resistance. Subsequently, women at risk for GDM or MetS should be actively informed, clinically examined, and treated when needed. In other words, the results demonstrate a possible way to interfere with the progression of MetS by improving oral health. However, further studies should be conducted in this respect, i.e., to evaluate the possibilities of oral health care on the overall metabolic balance among obese persons at high risk for MetS.

## Author Contributions

**Hanna Poulsen:** the design and the implementation of the study, literature search, writing, and editing. **Jukka Meurman:** writing, drafting, and editing the article, supervision. **Hannu Kautiainen:** data‐analysis. **Emilia Huvinen:** participated in the design of the original RADIEL study, editing. **Saila Koivusalo:** initiated, participated in the design of the original RADIEL study, coordinated the study. **Johan Eriksson:** writing, analysis of the results, editing, supervision. All authors have read and approved the final version of the manuscript.

## Conflicts of Interest

The authors declare no conflicts of interest.

## Data Availability

Data are available on request due to privacy/ethical restrictions. The data that support the findings of this study are available on request from the corresponding author. The data are not publicly available due to privacy or ethical restrictions.
